# Workplace Violence and Psychological Impact: A Study Over Two Decades

**DOI:** 10.1192/j.eurpsy.2025.1811

**Published:** 2025-08-26

**Authors:** K. Imen, R. Nakhli, L. S. Chaibi, A. Fki, N. Belhadj, M. Bouhoula, M. Maoua, H. Kalboussi, O. El Maalel, S. Chatti, A. Chouchane, A. Aloui, N. Mrizak

**Affiliations:** 1 Occupational Medicine Department, University of Sousse, Faculty of Medicine Ibn Al Jazzar, Sousse; 2 D Psychiatric Department, Razi Hospital, Tunis; 3 Occupational Medicine Department, Occupational Medicine Department, Sahloul University Hospital, Sousse, Tunisia

## Abstract

**Introduction:**

Workplace violence, particularly in the healthcare sector, is on the rise and severely affects the quality of life and performance of healthcare workers. The psychological repercussions of such violence are particularly concerning, making it imperative to develop appropriate prevention strategies.

**Objectives:**

To evaluate the prevalence of assaults against healthcare staff at the Farhat Hached University Hospital in Sousse over a 20-year period, describing the characteristics of the victims, the circumstances of the assaults, and the psychological impacts observed.

**Methods:**

This retrospective descriptive study, covering the years 2004 to 2023, analyzes workplace injury reports related to violence at the Farhat Hached University Hospital. The sample includes healthcare workers who were victims of reported assaults, with data collected on a synoptic sheet detailing sociodemographic aspects, the circumstances of the assaults, and their consequences.

**Results:**

Out of a total of 5695 workplace accidents, 110 were recorded as assaults, representing a prevalence of 1.9%. The victims had an average age of 46.8 ± 6.72 years, with a strong female predominance (69.1%). The most affected professions were nurses (50%) and workers (25.5%), with the most impacted departments being maternity (15.5%), emergency (13.6%), and psychiatry (11.8%). The majority of assaults (68.2%) occurred in the morning, and the perpetrators were primarily patients themselves (38.2%) or their relatives (33.6%).

Regarding the psychological aspects of the assaults, 42.7% of incidents involved psychological violence, such as verbal abuse, intimidation, or threats. The psychological consequences were significant: 26.4% of the victims developed disorders such as post-traumatic stress disorder (PTSD) and sleep disturbances.

**Conclusions:**

The high prevalence of assaults and their psychological repercussions highlight the urgent need to strengthen preventive measures. Specific training on conflict management and psychological support strategies should be implemented to improve the safety and well-being of healthcare staff.

**Disclosure of Interest:**

None Declared

